# Mental Health in Affectionate, Antagonistic, and Ambivalent Relationships During the COVID-19 Pandemic: A Latent Profile Analysis

**DOI:** 10.3389/fpsyg.2021.631615

**Published:** 2021-09-01

**Authors:** Jasmina Mehulić, Željka Kamenov

**Affiliations:** Department of Psychology, Faculty of Humanities and Social Sciences, University of Zagreb, Zagreb, Croatia

**Keywords:** COVID-19 pandemic, intimate relationships types, latent profile analysis, adult Croatian citizens, stress, depression, anxiety, dyadic coping

## Abstract

The ongoing coronavirus disease-2019 (COVID-19) pandemic presents an acute stressor affecting mental health. In these stressful times, intimate relationships functioning could serve as a protective or a risk factor to the well-being of partners. Adult Croatian citizens engaged in intimate relationships (*N* = 727) reported their relationship characteristics and assessed symptoms of depression, anxiety, and stress during the state lockdown in May 2020. Three relationship profiles based on variations in key relationship characteristics were identified using latent profile analysis. Profiles represented distinct relationship types described as affectionate, ambivalent, and antagonistic relationships. These relationship types differed in their levels of love and perception of humility, responsiveness, and behavior of the partner. Relationship type was associated with mental health symptoms such as depression, anxiety, and stress during the COVID-19 pandemic and state lockdown. Being in an affectionate relationship was associated with the lowest levels of depression, anxiety, and stress, while in an antagonistic relationship these are in the highest levels. Ambivalent relationships were characterized by moderate levels on all measured mental health indicators with no difference in anxiety compared with affectionate relationships. The results emphasized the link between relationship functioning and successful coping with mental health hazards such as the fear of disease or restrictive measures put in place to contain the COVID-19 pandemic.

## Introduction

The coronavirus disease-2019 (COVID-19) has affected the lives of many people in numerous and complex ways ever since its rapid spread throughout the world beginning in January 2020. Official data report shows that at his very moment there are 55,624,562 people affected by the SARS-Cov-2 virus, 35,800,000 people are cured, and 1,338,100 people have died (JHU CSSE, 2020). The pandemic has brought numerous changes in our way of living along with being a severe danger for the health of the people. Many countries took preventive measures in order to protect the lives of the people through closing schools and kindergartens, as well as bars, restaurants, and shopping malls. The culture and entertainment events were canceled, commuting was restricted, and people were asked to work from home, stay inside, and keep physical distance from other people (European Centre for Disease Prevention Control, 2021). This “lockdown” contributed to the deteriorating mental health of many people as much as it was perceived necessary and the only possible solution at the time.

In China, moderate and severe symptoms of anxiety, stress, and depression were found among the citizens as the disease started to threaten their health and lives. (Huang and Zhao, 2020). Comparable results were reported from Hong Kong where a quarter of surveyed participants declared impaired mental health with higher levels of depression and anxiety due to the COVID-19 pandemic (Choi et al., 2020). In the study of Ettman et al. (2020), they found that the adults from the United States of America (USA) showed a three-fold higher prevalence of depression symptoms during the pandemic than before. The COVID-19 pandemic brought numerous physical and mental health risks, which have been shown to result in moderate to severe depression, anxiety, and traumatic stress-related difficulties in the general population (Wang C. et al., 2020). Additionally, measures that governments undertook all over the world to prevent the spread of COVID-19 have both short- and long-term negative impacts on mental health and well-being (Brooks et al., 2020). Finally, the negative social and economic consequences of these measures are expected to be additional risk factors for mental health which may persist for a long time after the pandemic is over (Vukčević Marković et al., 2020).

Since the very beginning of the COVID-19 pandemic and the government-imposed state “lockdown,” Croatian couples started to worry about the negative impact of being “locked-in” together for a long and indefinite time on their relationship; they were also worried about the well-being of each partner. The fear of detrimental consequences of this forced togetherness and the lack of autonomy on close relationships was evident both in comics and jokes shared via social networks, as well as in serious articles and talk shows in the media. Partners being together 24/7 was thought to be a severe cause of stress and as a risk factor for personal well-being during the COVID-19 pandemic.

The need to belong is a fundamental human motive nonetheless; meaningful and enduring social relationships are essential to health and well-being (Baumeister and Leary, 1995; Slavich, 2020). Social connectedness impacts physical and mental health as shown in many studies (Holt-Lunstad et al., 2015). Research in the field of intimate relationships shows that married people tend to experience better physical and mental health, and lower mortality than single or divorced people (Amato, 2000; Frisch and Simonsen, 2013; Zissimopoulos et al., 2013). The quality of the relationship between partners was identified as a crucial factor in these associations. Whether a couple is married or not, managing a satisfying relationship promotes their personal and professional functioning, enhances their physical and mental health, and helps the development of their children (Cummings and Davies, 2002; Whisman and Uebelacker, 2006).

People need each other even more and rely on significant others to provide social support in times of stress and crisis, such as during this pandemic (Haslam and Reicher, 2006; Taylor, 2006). However, according to the vulnerability-stress-adaptation model (Karney and Bradbury, 1995), perceived stress lowers the capacity of a partner for constructive and adaptive reactions within the relationship (Baumeister, 2014; Neff and Karney, 2017); it also enhances the probability of maladaptive reactions (Neff and Karney, 2009). In turn, these hostile behaviors, negative communication, or even physical violence raise the level of experienced stress (Story and Bradbury, 2004; Bodenmann, 2005; Langer et al., 2008). For example, couples who reported elevated levels of perceived stress were less inclined to constructive problem-solving, which backfires on their level of perceived stress (Woszidlo and Segrin, 2013).

The study of Karney and Gauer (2010) suggests that in satisfying and stabilizing relationships, partners should be able to see things from the perspective of each other and avoid making maladaptive attributions for the behavior of each other. Recent evaluations of the construct humility show its value for maintaining quality, stability, and satisfaction in intimate relationships (Exline and Geyer, 2004). Interpersonal humility consists of modest self-presentation and an orientation toward others, which contribute to higher quality close relationships and better conflict resolutions (Wright et al., 2017). Humility is a positive affective state (Weidman et al., 2018) that includes prosocial and affiliative emotions and promotes understanding, forgiveness, and gratitude (Worthington et al., 2016). Individuals who perceive their partners as humble are more satisfied (Dwiwardani et al., 2018) and invest more in their relationships (Worthington et al., 2016). They also experience lower levels of stress (Ripley et al., 2016). Along with perceiving partners as humble, it is also beneficial for the relationship to perceive the partner as responsive to the needs of the other (Reis et al., 2004). Perceived responsiveness of the partner assumes a feeling of understanding, support, and respect of the other partner and contributes to more intimacy and satisfaction in a relationship (Reis and Shaver, 1988; Reis, 2017). It also promotes the well-being of partners, life satisfaction, and other positive effects (Gable et al., 2012; Otto et al., 2015).

These individual characteristics influence the way partners behave with each other and also shape their relationship (Kelley, 1979; Marshall et al., 2011). Behaviors have a huge role in affecting the quality of a relationship (Neff and Karney, 2009). In other words, the satisfaction of partners is reflected in the way they treat each other; in turn, it defines their satisfaction with their relationship, thus creating an interdependent system (Huston and Vangelisti, 1991; Neff and Karney, 2009). The studies of Caughlin and Huston (2006), as well as Vangelisti and Huston (1994), emphasized the importance of the interaction of partners with each other when identifying key domains of relationship satisfaction. Relationships can be described in terms of recurring behavioral processes that take place during an interaction between two partners, i.e., how often spouses criticize each other, how much they disclose, and how consistently they validate each other. Marital satisfaction, and warm or hostile behaviors of both partners start resembling each other as the time of the relationship progresses. All relevant theories in the field of intimate relationships assume such a cyclical relationship between behavior and relationship satisfaction and thus confirm the importance of specific behaviors as determinants of marital satisfaction. The *emotional climate* of a relationship reflects two core constructs: affection and antagonism. Combinations of the affectionate and antagonistic behaviors of partners in their everyday life differentiate relationships from one another (Caughlin and Huston, 2006). Affection and antagonism seem to be the two distinct dimensions, as confirmed by factor analyses and a low correlation between the two dimensions/factors (Smith et al., 1990; Huston and Vangelisti, 1991; Gable et al., 2003). In other words, lack of antagonism in a marriage still does not make it affectionate and happy, just as a lack of loving behaviors does not necessarily make it hostile. Affectionate and antagonistic behaviors often interact, and antagonistic behaviors can be interpreted differently depending on the level of affection they are embedded in Gottman (1994), Huston and Chorost (1994), Caughlin and Huston (2002), and Jelić et al. (2014). Therefore, the study of Caughlin and Huston (2006) suggests four archetypical emotional climates defined by the affection and antagonism dimensions: (1) high affection and low antagonism indicate *warm* marital climate, (2) high affection and high antagonism are typical of *tempestuous or stormy* emotional climate, (3) low affection and high antagonism represent *hostile* emotional climate in marriage, and (4) low affection and low antagonism characterize *bland* marriages. Marriages that fall somewhere between bland and tempestuous marriages are named *mixed blessings* in terms of emotional climate; it has an equal ratio of positive and negative elements.

Quality of interactions and communication between partners is sensitive to stress (Cutrona et al., 2003; Neff and Karney, 2004) and shows drastic decline even in experimental conditions (Bodenmann and Shantinath, 2004). External stressors and stressors within a relationship affect the quality of a relationship through the communication patterns between partners (Ledermann and Macho, 2009; Ledermann et al., 2010). Relational self-efficacy could be a protective factor in this process; individuals with higher relational self-efficacy are more prone to resolve conflicts through constructive communication in situations of perceived high levels of stress (Huić et al., 2016).

The traditional individual approach to stress and coping was challenged by many theorists at the beginning of the 1990s (Bodenmann et al., 2016). The systemic-transactional model (Bodenmann, 1995) being among the first model which suggested that perceived stress and coping are social processes embedded in close relationships. The model includes a focus on coping as a genuine dyadic phenomenon processed on the dyadic level. This dyadic level processing means that the stress signals of one partner and the coping reactions to these verbal and nonverbal signals of the other partner are taken into mutual consideration. In dependence upon the stress event, the stress management resources of both partners are activated in dyadic coping (DC) to maintain or restore a state of dyadic homeostasis (Bodenmann, 2005). Although people could cope with stress individually or with support from others, the quality of intimate relationships is affected through the reaction of one partner to the stress of the other partner as well as through joint DC in situations of mutual stressors such as the COVID-19 pandemic. Dyadic coping proved to be a predictor of relationship outcomes such as marital satisfaction and stability, quality of marriage, etc., as shown in the studies of Bodenmann and Cina (2006), Bodenmann et al. (2006), and Ledermann et al. (2010). The study of Bodenmann and Cina (2006) concluded that DC may significantly contribute to a strong feeling of “we-ness” within the couple by creating a cognitive internal working model of the relationship as being a helpful, supportive, enriching, and reliable resource.

## Current Study

The purpose of this study is to explore how different levels of key relationship variables: love, partner perception, partner's antagonistic and affectionate behavior, and DC form different relationship profiles. The study also aims to examine the association between different relationship profiles and mental health indices related to the COVID-19 pandemic: symptoms of depression, anxiety, and stress. Thus, the following research questions guided the study: What are the patterns of relationship characteristics? How many relationship profiles based on individual differences on key relationship variables can be distinguished using latent profile analysis (LPA)? Are these relationship profiles discriminately associated with depression, anxiety, and stress during the COVID-19 pandemic and the state lockdown?

Consistent with the exploratory nature of this study, we postulated no a priori hypotheses regarding the number of emerging profiles. Consequently, we did not formulate specific conjectures about the associations between specific profiles and mental health indices. However, we did presume more than one profile would emerge and expected participants in better functioning intimate relationships would show lower levels of depression, anxiety, and stress.

## Materials and Methods

### Participants and Procedures

Data were collected from May to June 2020 in Croatia as part of a larger *How are we?—Life in Croatia in time of the Corona pandemic* study focusing on the effects of the COVID-19 pandemic in Croatia, as well as a strong earthquake taking place in the Zagreb area in March 2020. Participants were recruited through newspapers, online portals, University mailing lists, word of mouth, and using the snowball recruitment method. The large study goal was to inquire about the changes in way of living, parenting, relationships, work, school, and stress levels caused by the newfound situation and to investigate the coping mechanisms of all generations. This was done through an online survey on the SoSciSurvey platform and with a specific questionnaire structure consisting of 10 separate but compatible branches. After having answered the questions in the common branch, participants could choose in which order they would like to access other branches. Only relevant branches were displayed based on the sociodemographic characteristics of the respondents.

Among the 4,029 persons who took the survey, 157 (3.9%) were excluded because they provided answers only for the sociodemographic questions. Out of the remaining 3,872 participants, 2,366 (70.7%) were in a relationship and 792 (33.5%) proceeded to complete the part of the questionnaire about relationship characteristics. Included in the 792 participants who took part in the intimate relationships branch, 727 (91.8%) provided answers to 50% or more of the questions. Thus, the analytical sample included 727 partnered individuals (*M*_age_ = 36.37, *SD* = 12.89, range 18–95 years; 85% of women). A multivariable logistic regression analysis was carried out with a binary outcome of having chosen to participate in the intimate relationship branch or not to address possible self-selection biases. Independent variables were age, gender, having children, and the level of completed formal education. Younger participants (AOR = 0.98, *p* < 0.001), with a higher level of completed formal education (AOR = 1.17, *p* = 0.001) and with children (AOR = 2.22, *p* < 0.001) had higher odds of taking part in the intimate relationship branch. Additionally, to address the possible biases introduced by excluding participants who answered less than half of all intimate relationship questions, another multivariable logistic regression was conducted with the binary outcome of having completed ≥50% of the branch items vs. having completed <50% of branch items. Independent variables were age, gender, having children, and the level of completed formal education. Participants had equally high odds of responding to at least half of the intimate relationship questions. The median time to complete the relationship branch of the questionnaire was 10–15 min.

Sample characteristics are shown in Table 1.

**Table 1:** Sociodemographic characteristics of the sample.

	***n* (%)^**a**^**
Gender	
Women	618 (85.1%)
Men	108 (14.9%)
Cohabiting with the partner	
Yes	481 (66.2%)
No	246 (33.8%)
Marital status	
Married	353 (48.6%)
Not married	373 (51.3%)
Having own children	
Yes	328 (45.1%)
No	399 (54.9%)
Number of children younger than 18 living in the household	
0	477 (65.6%)
1	99 (13.6%)
2	110 (15.1%)
3	35 (4.8%)
4 or more	6 (0.8%)
Type of relationship/marriage	
Heterosexual	692 (95.2%)
Homosexual	35 (4.8%)
Finished level of formal education	
Primary school	7 (1%)
High school	179 (24.6%)
College or Undergraduate	153 (21%)
Graduate (Master's Degree)	288 (39.6%)
Specialization, PhD	100 (13.8%)
Employment status	
Student	210 (28.9%)
Employed	414 (57%)
Unemployed	49 (6.7%)
Retired	25 (3.4%)
Parental leave	16 (2.2%)
Other	13 (2.8%)
	***M (SD)*** **in years**
Age	36.37 (12.89)
Relationship duration	10.04 (10.68)

a *Percentages do not always add up to 100 due to rounding up*.

More than three-quarters of all participants were women (85.1%). Women were somewhat younger (*M* = 35.37, *SD* = 12.42) than men (*M* = 42.18, *SD* = 14.05). The sample was heterogeneous regarding the education of the participants, with most participants having completed at least a bachelor's or a master's degree. Slightly over a half of the sample was employed (57%), almost one-third was still studying (28.9%) and the rest were unemployed (6.7%), retired (3.4%), or on parental leave (2.2%). Half of all participants reported being married (48.6%) and having children (45.1%). Approximately two-thirds were cohabiting with their partner at the time of the survey (66.2%) and living without children younger than 18 in their household (65.6%). The average relationship duration was 10 years (*SD* = 10.68, range 2 months−50 years). Only 4.8% of respondents reported being in a same-sex relationship.

### Measures

*Sociodemographic indicators* were gender, age, level of completed formal education, employment status, type of partnership/marriage (opposite-sex/same-sex), cohabiting, being married, having own children, and the number of children in the household. None of the sociodemographic indicators were used in further analyses aside from depicting the sample.

*Mental health* was measured with the 21-item Depression Anxiety Stress Scale (DASS; Lovibond and Lovibond, 1995), a set of three self-report scales designed to measure the emotional states of depression, anxiety, and stress. Answers were recorded on a 4-point scale ranging from 0 = “did not apply to me at all” to 3 = “applied to me very much or most of the time.” Items include statements such as “I felt that I had nothing to look forward to” or “I was worried about situations in which I might panic and make a fool of myself.” Sum scores for each subscale were computed by adding up the scores on all items per subscale and multiplying them by 2 with higher scale scores indicating more depressive, anxious, and stress symptoms, respectively. Internal consistency of each subscale was acceptable (Cronbach's α_depression_ = 0.92, α_anxiety_ = 0.90 and α_stress_ = 0.93). These subscales offer categorization of symptoms based on score range as follows: *normal functioning* ranging from 0 through 9; *mild symptoms* ranging from 10 through 13; *moderate symptoms* from 14 through 20; *severe symptoms* from 21 through 27; *extreme symptoms* with values above the value of 28.

*Love and intimacy* were conceptualized as the extent to which one feels a sense of closeness, belonging, and attachment to their partners corresponding with the construct of compassionate love (Hatfield and Rapson, 1993). Love and intimacy were assessed using the unidimensional 9-item Love Scale, a subscale from the Relationships Questionnaire (Braiker and Kelley, 1979) with items such as “To what extent do you love your partner?” and “How close do you feel toward your partner?” The item “How sexually intimate are you with your partner?” was excluded as it pertained to sexual behavior which was assessed with a different measure not included in this paper. The answers were anchored on a 9-point scale ranging from 1 = “not at all to 9 = “extremely.” A higher score indicates greater feelings of love for and belonging to the partner. Internal consistency was at Cronbach's α_love_ = 0.94.

*Perceived partner responsiveness*, the perception of a partner to the responsiveness, understanding, and validation of the other partner to themselves (Reis and Carmichael, 2006) was measured with eight items from the 12-item Perceived Partner Responsiveness Scale (PPRS; Reis et al., 2011) on a 7-point scale from 1 = “not at all true” through 4 = “moderately true” to 7 = “completely true.” Items include the stem “My partner usually:” with statements such as “really listens to me,” “seems interested in what I am thinking and feeling,” and “understands me.” The scale had high internal consistency in the current study (Cronbach's α = 0.96) with a higher composite score indicating higher perceived partner responsiveness.

*Perceived partner humility* was measured using an 11-item Perceived Partner Humility Scale (PPHS) (Mehulić et al., 2020) with answers anchored on a 7-point scale ranging from 1 = “not at all true, through 4 = “moderately true” to 7 = “completely true.” The items include statements such as: “He/she tries to understand others' perspective” and “Has an overly high opinion about himself/herself” The composite measure had acceptable internal consistency (Cronbach's α = 0.78) and was calculated as the average of the responses to all items, with a higher result indicating a higher level of perceived partner humility.

*Marital climate/socio-emotional behavior* in a relationship was measured using the Inventory of Affection and Antagonism in Marriage (Huston et al., 2010). The inventory measures affectionate and antagonistic partner behaviors in the past month. The affection of the partner was assessed with 8 items describing positive behaviors such as “Your partner did something nice for you that you didn't expect” and partner's antagonism was measured with 8 items describing their negative behaviors in a relationship such as “Your partner showed anger or impatience by yelling, snapping, or raising his/her voice at you” on a 5-point scale (1 = not once; 2 = once; 3 = two or three times; 4 = several times; 5 = regularly). Higher scores on the affection subscale indicate a higher frequency of affectionate behavior of a partner, and higher scores on the antagonism subscale indicate a higher frequency of antagonistic behavior of a partner. Internal consistency of both scales was adequate (Cronbach's α_affection_ = 0.91 and α_antagonism_ = 0.85).

*Dyadic coping*. Dyadic coping was measured using two subscales from the Dyadic Coping Inventory (DCI; Bodenmann, 2008). The DCI assesses different forms of DC, e.g., common, supportive, negative, and delegated DC, as perceived by oneself and as perceived by their partner. The first subscale used was the 5-item Common DC (CDC) measuring asymmetric or complementary involvement of both partners in a shared coping process expressed through talking about the stress and its meaning for each partner; jointly trying to reframe them and searching for more information, mutual efforts to calm down, or sharing emotional or physical intimacy (Bodenmann et al., 2016). The items include statements such as: “We try to cope with the problem together and search for ascertained solutions” with answers being anchored on a 5-point scale (1 = very rarely; 2 = rarely; 3 = sometimes; 4 = often; 5 = very often). The second subscale used was the 2-item Evaluation of Couple's DC with items such as “I am satisfied with the support I receive from my partner and the way we deal with stress together” and the answers are anchored on the same 5-point scale as above. Internal consistency of both scales was adequate (Cronbach's α_commondyadiccoping_ = 0.89 and α_evaluation_ = 0.93) with a higher score on both indicating greater common DC and a better evaluation of a couple's DC, respectively.

### Statistical Analyses

The missing values in the analytical sample (*N* = 727) were missing completely at random [Little's MCAR χ^2^ (952) = 980.57.82, *p* = 0.25] with most key variables having <1% of missing values except for the marital climate indicators (2–3% of missing values) and the DC indicators (5% of missing values). In the current study, to discover the number of emerging profiles of relationship functioning, we conducted an LPA, and to assess the associations between specific relationship profiles and mental health indices we conducted an analysis of variance. Additionally, using chi-square statistics we provided an insight into the prevalence of depression, anxiety, and stress categorized according to reported symptom intensity across relationship types. All statistical procedures were carried out using IBM SPSS 25 statistical software package and Mplus 8.

## Results

The results in Table 2 show that participants on average felt love and closeness (*M* = 7.63, *SD* = 1.45) toward their partner perceiving their partners as responsive (*M* = 5.43, *SD* = 1.4) and humble (*M* = 4.90, *SD* = 0.99) and their behavior in the past month as more often affectionate (*M* = 3.84, *SD* = 0.95) than antagonistic (*M* = 2.14, *SD* = 0.84). In times of stress, participants reported coping with it together with their partner (*M* = 3.69, *SD* = 0.96) and evaluated their joint coping positively (*M* = 3.88, *SD* = 1.09). No gender differences were observed on any of the key indicators apart from antagonistic behavior of a partner *t*_(710)_ = 2.12, *p* = 0.035; Cohen's *d* = 0.22). Men described the behavior of their partners in the past month as more antagonistic (*M* = 2.30, *SD* = 0.90) compared with women (*M* = 2.11, *SD* = 0.83). This overall positive evaluation of the partner and the relationships corresponds with the experience that individuals who are satisfied with their relationships tend to participate in studies assessing relationship characteristics (Karney and Bradbury, 2010).

**Table 2:** Descriptive statistics.

		***M* (*SD*)**	***t***
Love Scale	Men	7.60 (1.58)	−0.21
	Women	7.64 (1.43)	
	Total	7.63 (1.45)	
Perceived partner humility	Men	5.86 (0.87)	−0.42
	Women	4.90 (1.01)	
	Total	4.90 (0.99)	
Perceived partner responsiveness	Men	5.50 (1.34)	0.51
	Women	5.42 (1.42)	
	Total	5.43 (1.40)	
Partner's affectionate behavior	Men	3.80 (1.10)	−0.41
	Women	4.85 (0.92)	
	Total	3.84 (0.95)	
Partner's antagonistic behavior	Men	2.30 (0.90)	2.12^*^
	Women	2.11 (0.83)	
	Total	2.14 (0.84)	
Common dyadic coping	Men	3.65 (1.05)	−0.43
	Women	3.70 (0.95)	
	Total	3.69 (0.96)	
Evaluation of dyadic coping	Men	3.92 (1.12)	0.39
	Women	3.88 (1.08)	
	Total	3.88 (1.09)	
Depression	Men	8.20 (10.50)	−2.56^*^
	Women	10.98 (10.38)	
	Total	10.59 (10.45)	
Anxiety	Men	4.41 (6.92)	−4.59^***^
	Women	7.93 (9.44)	
	Total	7.42 (9.19)	
Stress	Men	9.83 (9.73)	−5.69^***^
	Women	16.28 (11.05)	
	Total	15.34 (11.11)	

* *p < 0.05;*

** *p < 0.01;*

*** *p < 0.001*.

The love of the participants for their partner was moderately associated with perceiving their partner as responsive (*r* = 0.75, *p* < 0.001) and humble (*r* = 0.54, *p* < 0.001), with the link between love and perceived partner humility being somewhat stronger for women (*r* = 0.57, *p* < 0.001), compared with men (*r* = 0.40, *p* < 0.001). The responsiveness of a partner was most strongly correlated with the affectionate (*r* = 0.80, *p* < 0.001) and antagonistic (*r* = −0.68, *p* < 0.001) behavior of a partner, as well as the evaluation of successful DC (*r* = 0.80, *p* < 0.001).

Finally, over a half of all participants reported experiencing no depressive (*N* = 404; 55.8%), anxious symptoms (*N* = 470; 64.7%) and stress (*N* = 390; 53.8%). On average, participants reported low anxiety (*M* = 7.42, *SD* = 9.19), being mildly depressive (*M* = 10.59, *SD* = 10.45) and moderately stressed (*M* = 15.34, *SD* = 11.11) with significant gender differences in all three mental health domains (*t*_[721]depression_ = −2.56, *p* = 0.01; *t*_[723]anxiety_ = −4.59, *p* < 0.001; *t*_[722]stress_ = −5.69, *p* < 0.001). Women reported more depressive (*M* = 10.98, *SD* = 10.38), anxious (*M* = 7.93, *SD* = 9.44), and stress (*M* = 16.28, *SD* = 11.405) symptoms compared with men (*M*_depression_ = 8.2, *SD* = 10.5; *M*_anxiety_ = 4.41, *SD* = 6.92; *M*_stress_ = 9.83, *SD* = 9.73). An increase in these symptoms was negatively associated with all relational variables except antagonism which correlated mildly and positively with depression (*r* = 0.22, *p* < 0.001), anxiety (*r* = 0.17, *p* < 0.001), and stress (*r* = 0.20, *p* < 0.001). The association between the antagonistic behavior and depressive symptoms of a partner was stronger for men (*r* = 0.27, *p* < 0.001) than women (*r* = 0.23, *p* < 0.001). Similarly, the association with anxiety was also stronger for men (*r* = 0.27, *p* < 0.001) than women (*r* = 0.17, *p* < 0.001) as was the association with stress (*r*_men_ = 0.34, *p* < 0.001; *r*_women_= 0.21, *p* < 0.001).

The empirically derived latent profiles were primarily established and then we examined associations of profiles with mental health subscales of the DASS using analysis of variance to ascertain the link between relationship functioning and the mental health of the participants.

### Latent Profile Analysis (LPA)

A mixture model technique called latent profile analysis (Oberski, 2016) was performed to identify subtypes of homogeneous latent classes or subgroups within a large heterogeneous group by obtaining the probability that individuals belong to different groups based on the similarity of the patterns of responses of the participants (Hagenaars and McCutcheon, 2002). The observed variables were continuous, composite scores of the Love Scale, PPRS, Perceived Partner Humility, marital climate subscales of Antagonism and Affectivity, CDC, and the Evaluation of Dyadic Coping (EDC) to identify relationship classes.

Model fit was assessed sequentially for one- through four-class models. Indicators were unstandardized, variances were freed to vary across profiles, and maximum likelihood estimates with robust standard errors (MLR) addressed missing data. Several fit criteria were used to determine the optimal number of profiles. Akaike's information criterion (AIC), Bayesian information criterion (BIC), and the sample-size adjusted BIC (SABIC) are goodness-of-fit-measures with lower values indicating a better fitting model (Muthén and Muthén, 2012). Entropy is the accuracy in assigning individuals to profiles, ranging from 0 to 1. The closer the value of entropy to 1, the more likely it is that individuals belong to the profile group they have been assigned to. Entropy values of 0.8 or greater indicate profile classification with minimal uncertainty (Tein et al., 2013). Additionally, The Bootstrap Likelihood Ratio Test (BLRT; McLachlan, 1987) and the Lo-Mendell-Rubin LRT Test (LRT; Lo et al., 2001) compared improvement between the fit of the estimated model compared with a more parsimonious model with one less profile (*k* – 1) and helped in assessing whether additional profiles were improving fit or discrimination of the model (Ferguson et al., 2020). Significant LMR and BLRT *p-*values suggest the more parsimonious model (with one less profile) is rejected in favor of the estimated model. Optimal models were chosen based on goodness of fit and parsimony.

The three-profile solution was retained as the model best-fitting to the data based on large decreases in AIC, BIC, and SABIC values until the difference between the three- and four-profile solution. The entropy value was greater than 0.8 for all models. Although the BLRT for the three-profile solution was significant indicating that the four-profile solution is a better representation compared with the three-profile solution, the LMR was not significant for the four-profile solution, supporting the more parsimonious model. The smallest class contained more than 5% of the sample, and the profiles were supported by the theory which makes it easier to justify and interpret (Ferguson and Hull, 2019). Summary of model selection indices of latent profile solutions for the total sample used to determine the optimal number of profiles is presented in Table 3.

**Table 3:** Summary of model selection indices of latent profile solutions for the total sample.

**Model**	**AIC**	**BIC**	**SABIC**	**Entropy**	**LMR *p***	**BLRT *p***	**Smallest class**	
							**% of *N***	**f**
1-Class	14,942.02	15,007.26	14,962.81					
2-Class	12,274.35	12,375.31	12,305.45	0.94	0.004	<0.001	24.21	176
**3-Class**	**11,295.87**	**11,433.54**	**11,338.28**	**0.93**	** <0.001**	** <0.001**	**11.55**	**84**
4-Class	11,020.50	11,194.80	11,074.22	0.89	0.39	<0.001	6.88	50

*N = 727; The LMR test and the BLRT compare the current model with a model with k – 1 profiles. AIC = Akaike's Information Criterion; BIC = Bayesian Information Criterion; SABIC = Sample-Adjusted BIC; LMR = Lo-Mendell Ruben; BLRT = bootstrap likelihood ratio test; f = n of individuals in the smallest class. Fit statistics for the best-fitting model are in boldface*.

**Figure 1:**
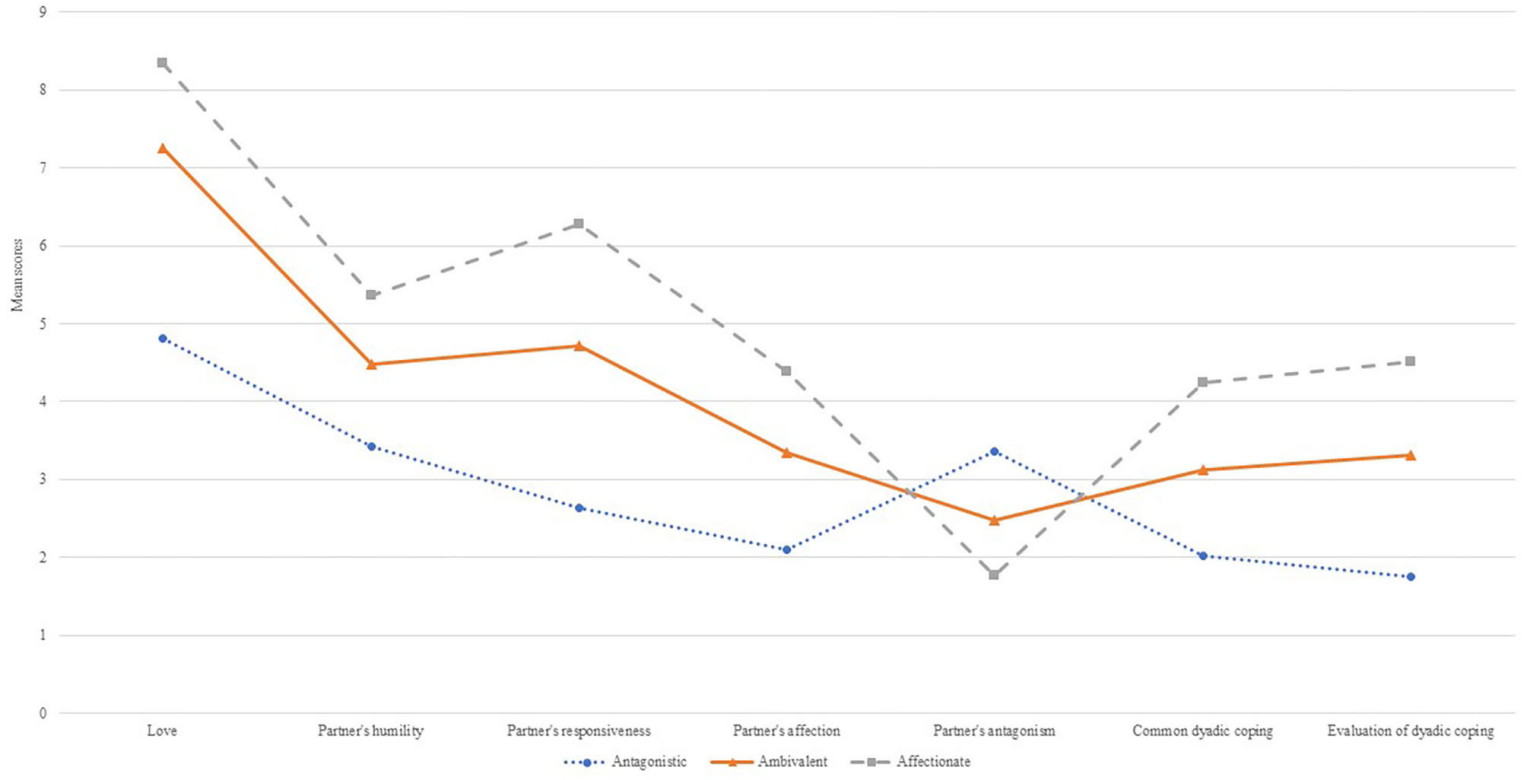
Profiles of the selected three-profile model.

Three profiles were identified representing antagonistic relationship, ambivalent relationship, and affectionate relationship types in the retained profile solution. The *M* and SD of variables used to create the chosen three-profile model are presented in Table 4 and Figure 1.

**Table 4:** Means and standard values for the three profiles.

**Variable**	**Profile 1**	**Profile 2**	**Profile 3**
	**Antagonistic relationships** **(*n* = 84)**	**Ambivalent relationships** **(*n* = 189)**	**Affectionate relationships** **(*n* = 454)**
Love	5.34 (0.34)	8.08 (0.40)	9.29 (0.40)
Perceived partner humility	4.65 (0.20)	6.10 (0.19)	7.30 (0.23)
Perceived partner responsiveness	3.86 (0.24)	6.93 (0.25)	9.22 (0.34)
Partner's affectionate behavior	3.83 (0.19)	6.12 (0.25)	8.03 (0.28)
Partner's antagonistic behavior	5.19 (0.28)	3.83 (0.15)	2.73 (0.08)
Common dyadic stress coping	3.47 (0.17)	5.36 (0.22)	7.29 (0.27)
Evaluation of Dyadic Coping	2.98 (0.21)	5.64 (0.34)	7.70 (0.38)

*N = 727*.

The differences between the three latent groups are mostly due to differences in all variables of interest: love, perceived partner responsiveness, perceived partner humility, affectionate and antagonistic behavior of a partner, and DC. The antagonistic relationship profile (*n* = 84, 11.55%) was characterized by the highest report of antagonistic behavior of the partner and the lowest perception of love, DC, and the partner being responsive and humble. The ambivalent relationships profile (*n* = 189, 26%) was characterized by similar perceptions of affectionate and antagonistic behavior of the partner. The affectionate relationship profile included the majority of participants (*n* = 454, 62.45%) and endorsed the highest mean levels of love, DC, perceived responsiveness and humility of the partner, affectionate behavior of the partner, as well as the lowest levels of antagonistic behavior the partner.

### Correlates of Profile Group Membership

Associations of profile group membership with depression, anxiety and stress level are presented in Table 5. Significant differences between profile groups were evident in the depression of participants, *F*_(2,721)_ = 22.31, *p* < 0.001; their anxiety, *F*_(2,723)_ = 6.74, *p* = 0.001; their stress, *F*_(2,722)_ = 25.85, *p* < 0.001. *Post hoc* analyses showed that participants in antagonistic relationships compared with those in ambivalent and affectionate relationships reported significantly higher levels of depressive (*p* = 0.001; *p* < 0.001), anxious (*p* = 0.043; *p* < 0.001), and stress (*p* = 0.013; *p* < 0.001) symptoms. Participants in ambivalent relationships reported higher lower levels of depressive (*p* = 0.005), and stress (*p* = 0.017) symptoms compared with those in affectionate relationships, however, there was no difference in their anxiety levels (*p* = 0.488).

**Table 5:** Means on DASS-21 for participants in three relationship types and statistics before the COVID-19 pandemic.

	**Antagonistic relationships** **(*n* = 84)**	**Ambivalent relationships** **(*n* = 189)**	**Affectionate relationships** **(*n* = 454)**	**Averaged across** **all profiles**	**February 2020^**b**^**
Depression	16.70 (11.54)	11.83 (10.93)	8.95 (9.53)	10.67 (10.45)	11.63
Anxiety	10.67 (10.67)	7.66 (8.70)^a^	6.72 (8.88)^a^	7.42 (9.19)	4.40
Stress	20.80 (11.27)	16.54 (10.98)	13.84 (10.78)	15.34 (11.11)	10.40

*N = 727;*

a *Means designated with the same superscript do not differ significantly based on post hoc comparisons. All other results differ significantly based on post hoc comparisons.*

b *(Jokić-Begić et al., 2020)*.

When categorized according to the reported symptom intensity, the prevalence of depression, anxiety, and stress experienced in affectionate relationships were the lowest with around two-thirds of participants reporting no depressive, anxiety, and stress symptoms and with <15% reporting severe or extreme symptomatology. Around half of participants in ambivalent relationships reported no depression, anxiety, and stress symptoms whereas up to 20% reported their symptoms being severe or extreme. Finally, around a third of participants in antagonistic relationships indicated experiencing no symptoms whereas another third reported experiencing severe or extreme depression, anxiety, and stress. These differences were significant [χ^2^_(8,724)_ = 44.940, *p* < 0.001] (Table 6).

**Table 6:** Prevalence of DASS-21 depression, anxiety, and stress levels in different relationship types.

		**Antagonistic relationships (*n* = 84)**	**Ambivalent relationships (*n* = 189)**	**Affectionate relationships (*n* = 454)**	**Total for all relationship types**
		*n_*observed*_* (*n_*expected*_, %* ^a^)			
Depression	Normal	23 (46.3, 27.7%)	95 (104.9, 50.5%)	286 (252.8, 63.1%)	404 (55.8%)
	Mild	14 (12, 16.9%)	28 (27.3, 14.9%)	63 (65.7, 13.9%)	105 (14.5%)
	Moderate	21 (11.7, 25.3%)	30 (26.5, 16%)	51 (63.8, 11.3%)	102 (14.1%)
	Severe	9 (4.8, 10.8%)	12 (10.9, 6.4%)	21 (26.3, 4.6%)	42 (5.8%)
	Extreme	16 (8.1, 19.3%)	23 (18.4, 12.2%)	32 (44.4, 7.1%)	71 (9.8%)
Anxiety	Normal	39 (54.4, 46.4%)	119 (122.4, 63%)	312 (293.3, 68.9%)	470 (64.7%)
	Mild	6 (5.6, 7.1%)	10 (12.5, 5.3%)	32 (30.0, 7.1%)	48 (6.6%)
	Moderate	14 (10.0, 16.7%)	29 (22.4, 15.3%)	43 (53.7, 9.5%)	86 (11.8%)
	Severe	9 (4.0, 10.7%)	11 (9.1, 5.8%)	15 (21.8, 3.3%)	35 (4.8%)
	Extreme	16 (10.1, 19%)	20 (22.6, 10.6%)	51 (54.3, 11.3%)	87 (12%)
Stress	Normal	29 (44.6, 34.9%)	87 (101.7, 46%)	274 (243.7, 60.5%)	390 (53.8%)
	Mild	11 (11.3, 13.3%)	32 (25.8, 16.9%)	56 (61.9, 12.4%)	99 (13.7%)
	Moderate	15 (10.9, 18.1%)	29 (24.8, 15.3%)	51 (59.4, 11.3%)	95 (13.1%)
	Severe	14 (8.7, 16.9%)	22 (19.8, 11.6%)	40 (47.5, 8.8%)	76 (10.5%)
	Extreme	14 (7.4, 16.9%)	19 (16.9, 10.1%)	32 (40.6, 7.1%)	65 (9%)

*N = 727.*

a *Percentages do not always add up to 100 due to rounding up. Numbers in parentheses indicate expected count and column percentages, respectively*.

## Discussion

The current study offers insight into how co-occurring relationship characteristics and various relationship types were differently linked to mental health outcomes, such as depression, anxiety, and stress. Relationship characteristics in this study include love, coping with stress, partner perception, and the behavior of the partner from different relationship types. The findings of the study propose profiles representing three relationship types among 727 partnered men and women who participated in the study. Most of the surveyed individuals who reported being in affectionate relationships were characterized by elevated levels of love, CDC, and the efficacy of DC. These respondents viewed their partners as responsive, humble and their behavior as high in affection, and low in antagonism. A pattern with moderate M values on all variables emerged among one-quarter of participants who reported being in ambivalent relationships. Ambivalent relationships revealed somewhat lower levels of love, CDC, the efficacy of DC, and perceived partner responsiveness and humility compared with affectionate relationships. The most distinctive characteristics between these two types of relationships are the levels of perceived affectionate and antagonistic behaviors of the partner; individuals in ambivalent relationships reported similar levels of both behaviors. The remaining 11.55% of respondents were in antagonistic relationships characterized by low levels of love and positive behavior with the highest rates of having experienced an antagonistic behavior of a partner. This three-profile solution is comparable to the marriage typology of Caughlin and Huston (2006) based on the relational socioemotional climate characterized by affectionate and antagonistic behavior. According to Caughlin and Huston (2006), warm marriages correspond with our affectionate relationships, hostile marriages with our antagonistic relationships, and mixed blessings marriages with our ambivalent relationships.

Comparison of mental health indices between these three relationship types showed significant differences regarding depression, anxiety, and stress. Affective relationships were associated with the best mental health functioning compared with ambivalent and antagonistic relationships. Participants in antagonistic relationships reported the worst results on depression, anxiety, and stress scales.

The association between relationship functioning and individual well-being as viewed through depression, anxiety, and stress symptoms could be bidirectional. On one hand, these findings could indicate that good relationships serve as a protective factor and poor relationships as a risk factor for mental health functioning in times of severe external stress, such as the one caused by the COVID-19 pandemic. On the other hand, the same findings could suggest that individuals whose mental health was more strongly affected by the COVID-19 pandemic perceive their relationship and their partners in a more negative light or even act in a way that harms the quality of their relationships.

Firstly, relational well-being enhances individual well-being which is consistent with the Ryff and Singer (2000) *interpersonal flourishing perspective*. Quality relationships are associated with future life satisfaction (Be et al., 2013) and people who describe their relationships as closer and more intimate are happier, more satisfied, and report better mental and physical health (Mastekaasa, 1994). In line with this, bad relationships characterized by more prominent antagonistic behaviors and poor communication may increase perceived stress (Story and Bradbury, 2004; Bodenmann, 2005; Langer et al., 2008) through deteriorating the physical (Wickrama et al., 1997) and psychological well-being of a person (Coyne and DeLongis, 1986; Beach et al., 1998). Marital discord is significantly associated with an increased likelihood of depression (Beach et al., 2003), anxiety, and suicidal ideation (Santini et al., 2015). Additionally, poorly functioning relationships do not help buffer against acute and chronic external stressors because they lack affectionate behaviors that serve as a protective factor for the relationship and individual well-being (Conger et al., 1990). Satisfying relational and marital functioning protects against the development of psychological distress (Trudel and Goldfarb, 2010). In such satisfying relationships, partners can complement each other in terms of the resources for coping with stress (Bodenmann, 2005). When dealing with issues affecting both partners such as the COVID-19 pandemic, engaging in CDC, e.g., joint problem solving, joint information seeking, sharing of feelings, or relaxing together, alleviates negative stress impacts and also strengthens mutual trust and intimacy that further improve the relationship regardless of gender, age, relationship duration, education, and ethnicity (Falconier et al., 2015).

Secondly, individuals with psychological difficulties such as depression or anxiety or those reporting elevated levels of stress find themselves in relationships with poorer functioning (Schnapp et al., 2020). The individual distress of partners is related to relationship quality and satisfaction (Bahun and Huić, 2017), poorer communication (Williamson et al., 2013), lowered capacities for relationship maintenance (Buck and Neff, 2012), a stronger tendency towards emotional and physical aggression (Langer et al., 2008), and an overall negative representation of the relationship (Neff and Karney, 2004). Depressed individuals tend to engage in maladaptive cognitive coping strategies such as rumination, negative metacognitive beliefs, self-blaming, and wishful thinking (Billings and Moos, 1984; Papageorigou and Wells, 2003). Such depression is associated with poorer dyadic interactions (Bodenmann et al., 2004). Furthermore, depression is associated with dysfunctional individual coping resources and deficits in stress communication and DC (Bodenmann et al., 2004) which has an effect on their relationship quality as well as that of their partner. Relationship functioning and mental health mutually and constantly affect each other.

The results of the study reveal that a considerable proportion of the participants show a deterioration in their mental health as expected. Participants report more symptoms of depression and stress compared with symptoms of anxiety as indicated from their depression, anxiety, and stress levels. The prevalence of depression and stress symptoms in this study is comparable to those in other COVID-19 related studies whereas anxiety levels differ (Khan et al., 2020; Shah et al., 2020; Traunmüller et al., 2020). More specifically, the anxiety levels of participants in this study were lower than that of participants in other studies.

Inspite all that COVID-19 related studies show an increase in depression, anxiety, and stress symptoms, specific results and percentages differ from study to study based on the time of data collection, sample characteristics, and the overall social situation in any given country (e.g., Khan et al., 2020; Ozamiz-Etxebarria et al., 2020; Özdin and Bayrak Özdin, 2020; Shah et al., 2020; Traunmüller et al., 2020; Wang Y. et al., 2020). Thus, the anxiety levels in this study could have been lower compared with other COVID-19 related studies due to the broader social context in which data collection took place. Participants took part in this study at the time of decrease in daily infection rates during the lockdown and after having been “locked-in” for the previous 2 months. At such a time it seemed the disease was or would soon be under control which could have lowered anxiety levels of Croatian citizens. Given lowered anxiety levels, the variability of this variable could have been reduced, potentially explaining the fact that no significant difference in anxiety between affectionate and ambivalent relationship types was found.

Overall, the results of the study highlighted the link between relationship functioning and mental health symptomatology in times of severe external stressors such as the COVID-19 pandemic and pointed out a potential area for clinical interventions. Strategies for improving relationship functioning could enable partners to co-create relationships that could serve as a protective factor in these stressful times and promote more positive health outcomes. Concurrently, organized psychosocial assistance focused on managing mental health difficulties during the COVID-19 pandemic could prove helpful to maintaining satisfying and affectionate intimate relationships which could in turn further improve the well-being of the citizens.

## Limitations

Several limitations to our findings should be noted. Although we identified three relationship types using theoretically supported variables, we have focused on the experiences of only one partner in the dyad. Due to the interdependence between the partners in a relationship, future studies should include the other partner into the analysis attending to the dyadic context of people's lives to promote further understanding of mental health risks of the pandemic and their association with relationship functioning. Additionally, most of the sample were heterosexual women which makes the interpretation of the findings limited to that subset of the population. Due to a disproportion between men and women in the sample we were unable to inquire into possible gender differences in relationship types and mental health indicators. This should be addressed in future studies. Finally, the correlational and cross-sectional nature of this research provides no insight into the direction of the association between relationship functioning and mental health outcomes or change over time and the self-report nature of this research provides no biological information. Future researchers should consider other research designs to add a deeper understanding of the topic.

## Conclusion

The present study used LPA to identify different relationship types based on key relational variables such as love, partner perception, partner behavior, and DC. Additionally, the association between these relationship types and mental health symptoms related to the COVID-19 pandemic, such as depression, anxiety, and stress was examined. Three relationship types were identified and named antagonistic, ambivalent, and affectionate relationships. Results suggest couples in antagonistic relationships are at most risk of mental health problems compared with those in ambivalent and affectionate relationships. The affectionate relationship membership has been associated with the lowest levels of depression, anxiety, and stress. The results emphasized the link between relationship functioning and successful coping with severe external stressors that could endanger mental health.

## Data Availability Statement

The raw data supporting the conclusions of this article will be made available by the authors, without undue reservation.

## Ethics Statement

The studies involving human participants were reviewed and approved by the Institutional Review Board in Croatia at the Department of Psychology, Faculty of Humanities and Social Sciences, University of Zagreb. The participants provided their informed consent to participate in this study electronically.

## Author Contributions

Both authors contributed equally to the conception and the design of this work. Both authors collected the data, critically reviewed, and approved the final submitted version of the manuscript. JM: data processing, modeling, interpreting data, and manuscript writing. ŽK: interpreting data and manuscript writing. All authors contributed to the article and approved the submitted version.

## Conflict of Interest

The authors declare that the research was conducted in the absence of any commercial or financial relationships that could be construed as a potential conflict of interest.

## Publisher's Note

All claims expressed in this article are solely those of the authors and do not necessarily represent those of their affiliated organizations, or those of the publisher, the editors and the reviewers. Any product that may be evaluated in this article, or claim that may be made by its manufacturer, is not guaranteed or endorsed by the publisher.
